# The Y137H mutation of *VvCYP51* gene confers the reduced sensitivity to tebuconazole in *Villosiclava virens*

**DOI:** 10.1038/srep17575

**Published:** 2015-12-03

**Authors:** Fei Wang, Yang Lin, Wei-Xiao Yin, You-Liang Peng, Guido Schnabel, Jun-Bin Huang, Chao-Xi Luo

**Affiliations:** 1 Department of Plant Protection, College of Plant Science and Technology and the Key Lab of Crop Disease Monitoring & Safety Control in Hubei Province, Huazhong Agricultural University, Wuhan 430070, China; 2 Department of Plant Pathology, College of Agriculture and Biotechnology, China Agricultural University, Beijing 100193, China; 3 Department of Agricultural and Environmental Sciences, Clemson University, Clemson, SC 29634, USA

## Abstract

Management of rice false smut disease caused by *Villosiclava virens* is dependent on demethylation inhibitor (DMI) fungicides. Investigation of molecular mechanisms of resistance is therefore of upmost importance. In this study the gene encoding the target protein for DMI fungicides (*VvCYP51*) was cloned and investigated. The *VvCYP51* gene in the resistant mutant revealed both a change from tyrosine to histidine at position 137 (Y137H) and elevated gene expression compared to the parental isolate. In order to determine which of these mechanisms was responsible for the reduced sensitivity to DMI fungicide tebuconazole, transformants expressing the mutated or the wild type *VvCYP51* gene were generated. Transformants carrying the mutated gene were more resistant to tebuconazole compared to control transformants lacking the mutation, but the expression of the *VvCYP51* gene was not significantly correlated with EC_50_ values. The wild type VvCYP51 protein exhibited stronger affinity for tebuconazole compared to the VvCYP51/Y137H in both molecular docking analysis and experimental binding assays. The UV-generated mutant as well as transformants expressing the VvCYP51/Y137H did not exhibit significant fitness penalties based on mycelial growth and spore germination, suggesting that isolates resistant to DMI fungicides based on the Y137H mutation may develop and be competitive in the field.

*Villosiclava virens* (anamorph: *Ustilaginoidea virens*) is an ascomycete fungus causing rice false smut, a serious disease of rice cultivated around the world^1^,^2^,^3^,^4^. Currently, rice false smut is managed by routine applications of fungicides. Sterol demethylation inhibitors (DMIs) are among the most effective fungicides for false smut control and are widely used in China^5^. DMI fungicides bind to the heme iron of the cytochrome P450 sterol 14α-demethylase (CYP51) and thus interfere with the biosynthesis of ergosterol, the primary sterol in fungal membranes^6^. Consecutive applications of DMI fungicides have led to the emergence and selection of resistance in several plant pathogenic fungi^7^,^8^,^9^,^10^. With regard to the *V. virens*, no fungicide sensitivity data have been published and to the best of our knowledge no evidence of resistance to DMI fungicides has been reported.

Resistance to DMI fungicides most often is based on overexpression of or point mutations in the *CYP51* gene. Constitutive overexpression of the *CYP51* gene has been shown to cause DMI resistance in many plant pathogenic fungi^10^,^11^,^12^,^13^,^14^,^15^,^16^,^17^, whereas point mutations were only reported in some pathogens^18^,^19^,^20^,^21^,^22^. Other resistance mechanisms include increased expression of ATP-binding cassette (ABC) transporters and major facilitator superfamily (MFS) transporters encoding efflux pumps^23^,^24^,^25^.

The goal of this study was to investigate potential resistance mechanisms in *V. virens*. Because DMI-resistant isolates were not available, we produced a DMI-resistant mutant using UV-mutagenesis and cloned and sequenced the 14α-demethylase gene (designated as *VvCYP51*) from *V. virens*. Specific objectives were to (i) determine the variation of *VvCYP51* gene sequences and expression patterns between the UV-generated mutant and the parental isolate; (ii) investigate the role of the mutated gene through genetic transformation; (iii) and elucidate the affinity of DMI fungicide tebuconazole with VvCYP51 protein through molecular docking analysis and binding assays.

## Results

### Cloning the *VvCYP51* gene

**Figure 1:**
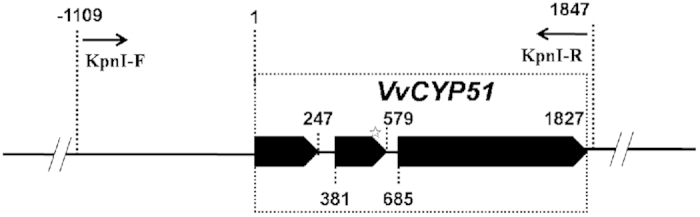
Schematic diagram of the promoter and coding region of the *VvCYP51* gene. The dotted box represents the entire *VvCYP51* gene which contains three exons indicated by the solid arrows and two introns indicated by solid lines between the exons. The asterisk represents the mutated site in the second exon of *VvCYP51* gene. The positions of primers used for transformation are indicated in the diagrams.

The alignment of all fragments obtained by inverse PCR from DNA of the isolate UV-8a was 4994 bp in length, encompassing the full-length *VvCYP51* gene (1827 bp) as well as upstream (2347 bp) and downstream (820 bp) flanking sequences. The entire *VvCYP51* gene of the isolate FJ4-1b was also amplified and revealed identical nucleotide sequences. The cDNA of the *VvCYP51* gene was synthesized from FJ4-1b RNA using primer pair RT-F/RT-R to determine the arrangement of exons. Comparison of the sequences of genomic DNA and cDNA revealed that the *VvCYP51* gene was 1827 bp in length containing three exons and two introns (Fig. 1). The full length cDNA was 1,587 bp in length and encoded a putative polypeptide of 528 amino acids. The *VvCYP51* gene sequence from UV-8a was deposited in GenBank (accession no. KJ004673).

**Figure 2:**
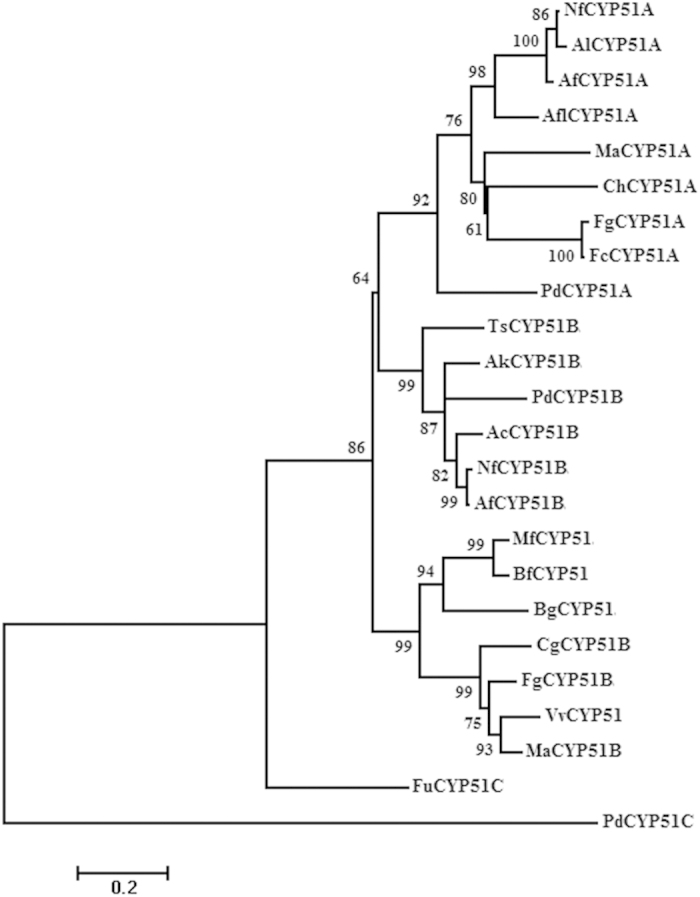
Phylogenetic tree generated by the maximum likelihood method with Mega 5.2 software on the basis of deduced amino acid sequences of CYP51. Sequences included that from *V. virens* isolate FJ4-1b, and those from other fungal species *M. anisopliae* (MaCYP51B, GenBank accession no. EFZ00272.1; MaCYP51A, EFZ04268.1), *F. graminearum* (FgCYP51B, ACL93392.1; FgCYP51A, AFN6619.1), *Colletotrichum gloeosporioides* (CgCYP51B, ELA23688.1), *B. graminis* (BgCYP51, AF052515.1), *M. fructicola* (MfCYP51, ACY41222.1), *B. fuckeliana* (BfCYP51, CCD54835.1), *Talaromyces stipitatus* (TsCYP51B, XP_002478695.1), *P. digitatum* (PdCYP51B, AEK21498.1; PdCYP51C, AEK21497.1; PdCYP51A, EKV08007.1), *Aspergillus kawachii* (AkCYP51B, GAA89598.1), *Aspergillus clavatus* (AcCYP51B, XP_001273214.1), *Neosartorya fischeri* (NfCYP51B, XP_001261295.1; NfCYP51A, XP_001267338.1), *A. fumigates* (AfCYP51B, AF338660.1; AfCYP51A, AF338659.1), *F. ussurianum* (FuCYP51C, AGC81882.1), *Fusarium cerealis* (FcCYP51A, AFN66168.1), *C. higginsianum* (ChCYP51A, CCF38358.1), *Aspergillus flavus* (AflCYP51A, XP_002375123.1), *Aspergillus lentulus* (AlCYP51A, ADI80344.1).

Phylogenetic analysis of predicted amino acid sequences of CYP51 proteins, including the VvCYP51, was performed with the maximum likelihood method using MEGA 5.2 software. Results showed that VvCYP51 was homologous to the CYP51B protein from multiple other fungi (Fig. 2). The deduced amino acid sequence of VvCYP51 was 86% identical to that of *Metarhizium anisopliae* (MaCYP51B, GenBank accession no. EFZ00272.1), 83% identical to that of *Fusarium graminearum* (FgCYP51B, ACL93392.1), 68% identical to that of *Botryotinia fuckeliana* (BfCYP51, CCD54835.1) and *Monilinia fructicola* (MfCYP51, ACY41222.1). The percentage identity confirmed VvCYP51 to be a member of the fungal CYP51 family.

### Generation of a mutant with reduced sensitivity to tebuconazole

Conidial spores of the isolate FJ4-1b were treated by UV irradiation, only one of the UV treatments yielded a mutant that could grow on PSA containing 0.5 μg/ml tebuconazole. This mutant was designated as UV10th, because it grew for 10 generations on PSA amended with 0.5 μg/ml tebuconazole. The EC_50_ value of the mutant UV10th for tebuconazole was 0.22 μg/ml with the resistance factor (EC_50_ value of the mutant divided by the EC_50_ value of the parental isolate) of 5.12 compared to the wild-type parental isolate FJ4-1b (Table 1).

Alignment of *VvCYP51* gene cDNA sequences from isolate FJ4-1b and mutant UV10th showed a thymine (T) to cytosine (C) exchange at nucleotide position 409 (amino acid position at 137, Y137H). The relative expression of the *VvCYP51* gene was up to 55-fold increased in the UV10th mutant compared to the isolate FJ4-1b (Table 1).

### The Y137H mutation conferred reduced sensitivity to tebuconazole

**Figure 3:**
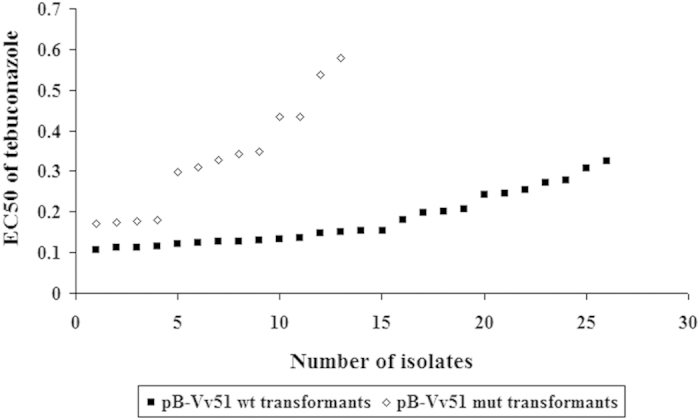
Sensitivity (EC_50_ values) of pB-Vv51wt and pB-Vv51mut transformants to tebuconazole. The isolates are separated by the number. The EC_50_ values were significant different between pB-Vv51wt and pB-Vv51mut transformants.

Twenty-six pB-Vv51wt transformants transformed with the *VvCYP51* gene and 13 pB-Vv51mut transformants transformed with the mutated *VvCYP51* gene (Y137H) were obtained to assess the relationship between the point mutation Y137H and the reduced sensitivity to tebuconazole. The results showed a significant increase (P < 0.01) of EC_50_ values for pB-Vv51mut transformants (average EC_50_ value of 0.33 μg/ml) compared to EC_50_ values for pB-Vv51wt transformants (average of 0.18 μg/ml), indicating that the point mutation Y137H was responsible for the reduced sensitivity to tebuconazole (Fig. 3).

### Overexpression was not a determinant of the reduced sensitivity

The expression of *VvCYP51* gene in the wild type isolate FJ4-1b was investigated after exposure to 0.1 μg/ml tebuconazole 6 h before RNA extraction. Results showed that the expression of *VvCYP51* gene increased 29.94-fold in isolate FJ4-1b compared to the corresponding, untreated control.

**Figure 4:**
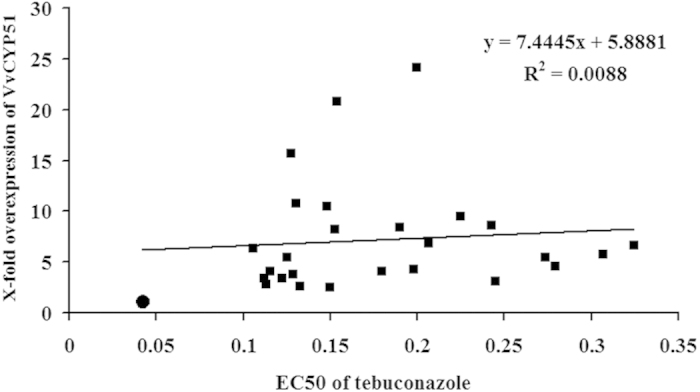
Relationships between relative expression of *VvCYP51* and sensitivity (EC_50_ values) to tebuconazole in tranformants. Linear regression analyses and coefficients of determination (R^2^) are shown no correlation. The circle denotes FJ4-1b and squares denote pBHt2-Vv51wt transformants.

To investigate whether overexpression of the *VvCYP51* gene was able to confer the reduced sensitivity to tebuconazole, the expression of the gene was determined in 26 pB-Vv51wt transformants and in parental isolate FJ4-1b. Different levels of relative expression ranging from 2.57 to 24.23 were observed (Table 1). EC_50_ values of the 26 pB-Vv51wt transformants ranged between 0.11 and 0.32 μg/ml and resistance factors ranged between 2.52 and 7.71 (Table 1). According to linear regression analysis, no correlation was observed between the *VvCYP51* expression levels and tebuconazole sensitivity (Fig. 4).

### Fitness of mutant/transformants containing the Y137H mutation

Comparison of mycelial growth rate, sporulation and spore germination showed no significant differences between the UV-mutagenesis isolate UV10th and the wild type isolate FJ4-1b (Table 2). However, some fitness parameters indicated a fitness cost in pB-Vv51mut transformants. The average mycelial growth rate of pB-Vv51mut transformants (4.0 mm/day) was significantly higher than that of pB-Vv51wt transformants (P < 0.05), but sporulation was significantly reduced (P < 0.05) (Table 2). Spore germination ability was not different among mutant/transformants.

### Molecular docking

**Figure 5:**
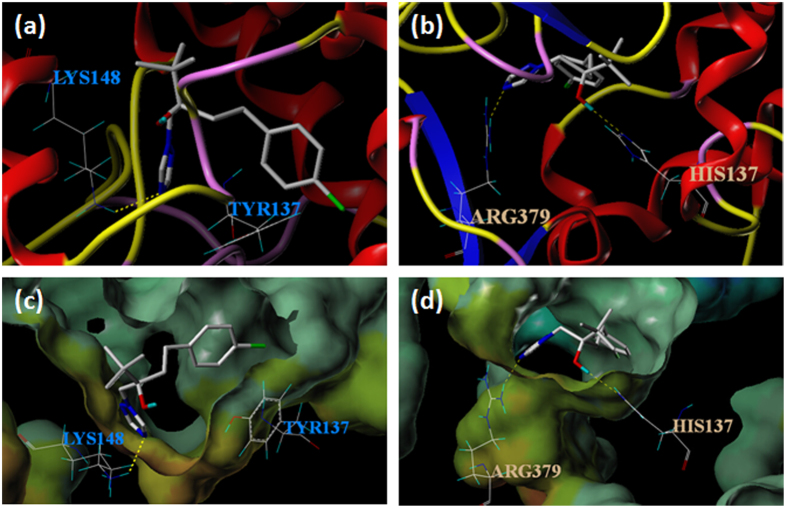
Molecule docking for the VvCYP51-tebuconazole complex. (**a**) Tebuconazole formed hydrogen bond (dotted line) with amino acid Lys148 in VvCYP51. (**b**) Tebuconazole formed hydrogen bond (dotted lines) with amino acids Arg379 and His137 in VvCYP51 with Y137H. (**c**) The hydrophobic and electrostatic environment of binding between Lys148 and VvCYP51. (**d**) The hydrophobic and electrostatic environment of binding between Arg379, His137 and VvCYP51 with Y137H. The color range for hydrophobicity potential ranges from brown (highest lipophilic area of the molecule) to blue (highest hydrophilic area). Electrostatic potential ranges from red (most positive) to purple (most negative). The colors of the environment in (**c,d**) did not change significantly from green and beige to blue or brown, indicating that the point mutation did not significantly change the hydrophobic and electrostatic environment of binding.

SYBYL X-2.0 software was used to determine the binding site and simulate the binding mode of the protein (VvCYP51) and ligand (tebuconazole). Tebuconazole formed a hydrogen bond with amino acid Lys148 of VvCYP51 in the wild type isolate FJ4-1b, but established contact with amino acids Arg379 and His137 in the mutant UV10th (Fig. 5). The point mutation of Y137H did not change the hydrophobic and electrostatic environment of binding (Fig. 5). The higher total score of H binding between tebuconazole and VvCYP51 compared to that between tebuconazole and VvCYP51/Y137H (total score 5.4982 vs 4.4795) indicated that tebuconazole interacted stronger with the isolate FJ4-1b than with the mutant UV10th (Table 3). The distance of centroids of tebuconazole and VvCYP51 (6.649) was less than that between tebuconazole and VvCYP51/Y137H (6.898), suggesting that there was a stronger affinity between tebuconazole and wild type VvCYP51 (Table 3). Docking analysis indicates that compounds showing less binding energy had higher inhibitory activity and, inhibitory constant is an index to measure compound inhibitory potency for a biological or biochemical function. Here binding energy (VvCPY51, −6.93; VvCYP51/Y137H, −6.63) and inhibitory constant (VvCYP51, 8.27; VvCYP51/Y137H, 13.72) both showed that the Y137H mutation decreased the binding tightness between VvCYP51 and tebuconazole (Table 4).

### Spectral analysis of tebuconazole binding to VvCYP51

**Figure 6:**
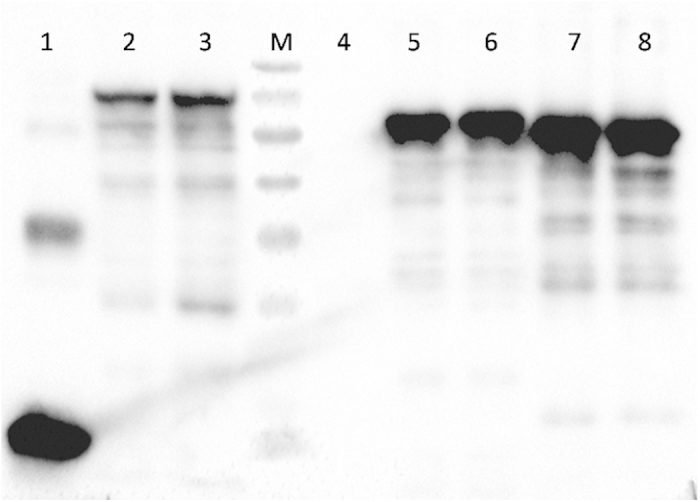
Western blot analysis of different truncated VvCYP51. M, protein marker. The largest to smallest bands are 95, 72, 55, 43, 34, 26, and 17 kDa in length. Lanes 1 to 8 represent transformants pET32a, FJ4-28aa, UV10th-28aa, pET28a, FJ4-42aa, UV10th-42aa, FJ4-74aa, UV10th-74aa, respectively.

The VvCYP51 protein formed inclusion bodies in all *E. coli* transformants. Western blot analysis indicated that the immunoreactive bands corresponding to the truncated 28-, 42- and 74 aa VvCYP51 proteins were consistent with the predicted molecular masses of 56.09, 54.68 and 51.02 kDa, respectively. The expression of 74 aa truncated N-terminal VvCYP51 was higher than that of 28- and 42 aa truncated VvCYP51 proteins (Fig. 6).

**Figure 7:**
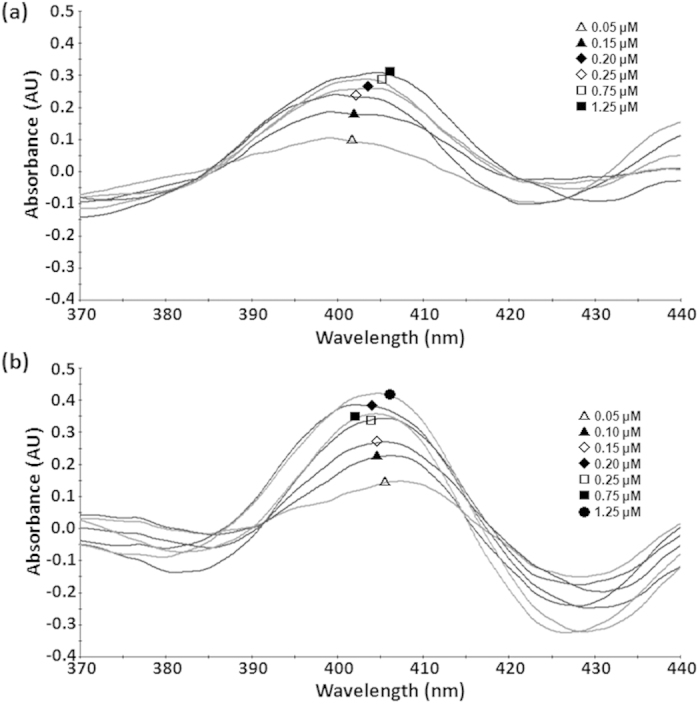
The absorption spectra of tebuconazole binding to 28aa truncated VvCYP51. (**a**) Truncated protein from FJ4-1b, (**b**) truncated protein from UV10th.

According to spectral analysis, the absorbance peak and trough were at 395–405 and 375–385 nm, respectively. The absorption spectra of 28 aa truncated VvCYP51 are shown in Fig. 7A (wild type) and 7B (VvCYP51/Y137H mutation) as an example. The absorption difference increased with fungicide concentration (Fig. 7). The control experiments (pET28a, pET32a with tebuconazole) did not show the dose-dependent variation (data not shown). The equilibrium binding constant (*K*_d_) is the ligand concentration required to obtain half the maximum binding at equilibrium. The calculated *K*_d_ values of three different truncated wild type VvCYP51 proteins (VvCYP51, less than 0.04 μM) were significantly lower than those of three truncated mutant VvCYP51 proteins (VvCYP51/Y137H; more than 0.07 μM), respectively (Table 3), suggesting that tebuconazole had higher affinity for wild type VvCYP51 than for VvCYP51/Y137H. Thus, the role of Y137H mutation in the mutant UV10th for the reduced sensitivity to tebuconazole was experimentally supported through decreased affinity of VvCYP51 protein to the tebuconazole molecule.

## Discussion

The enzyme CYP51 catalyses 14α-demethylation of sterols, but the copy number of *CYP51* gene is different in different species. Mammalian genomes always contain one *CYP51* gene^26^,^27^. The erg11/*CYP51* gene in *Saccharomyces cerevisiae* and *Candida albicans* is also single-copy^28^. Some fungi contain multiple *CYP51* genes, e.g., two in *Aspergillus fumigates* and *Magnaporthe oryzae*^29^,^30^, three in *Penicillium digitatum* and *F. graminearum*^11^,^31^. Based on the local BLAST analysis of *CYP51* genes from two *V. virens-*close species *M. anisopliae* (*CYP51A*, *CYP51B*) and *Fusarium ussurianum* (*CYP51C*) in the *V. virens* database, the *VvCYP51* gene was confirmed to be present as a single copy in the *V. virens* genome (http://www.ncbi.nlm.nih.gov/Traces/wgs/?val=JHTR01).

Homologous *CYP51* genes including *CYP51A*, *CYP51B*, and *CYP51C* have been found in some fungi and each may exhibit a different degree of fungicide resistance. The *CYP51A* gene is most commonly encoding the prime target of DMI fungicides, but the function of *CYP51B* is not consistently associated with resistance. For example, the sensitivity of *F. graminearum* mutants with *CYP51A* and *CYP51C* deletions to DMI fungicides increased, but there was no change in DMI sensitivity when *CYP51B* was deleted^31^; the *CYP51A* deletion mutants of *M. oryzae* were highly sensitive to DMI fungicides, while *CYP51B* deletion mutants were not altered in their sensitivity^30^. Contrary to the above, *CYP51B* of *P. digitatum* was shown to be involved in DMI fungicide resistance^11^; overexpression of the *ShCYP51B* gene in *Sclerotinia homoeocarpa* was associated with DMI resistance^25^. In the present study, *VvCYP51* was identified to genetically be most closely related to *CYP51B* genes according to phylogenetic analysis. While previous studies associated overexpression of *CYP51B* with resistance to DMI fungicides, this study showed that a single point mutation rather than overexpression was associated with the reduced sensitivity.

Substitutions of amino acids are often found in CYP51 proteins of lab mutants and field isolates of plant pathogenic fungi, such as Y136F in *Blumeria graminis*^18^, *M. fructicola*^20^, *Mycosphaerella fijiensis*^21^ and *Uncinula necator*^22^, Y137F in *Mycosphaerella graminicola*^32^,^33^. As mentioned above, the amino acids generally change from tyrosine to phenylalanine. These mutations alone or combined with other mutations conferred decreased sensitivity to DMI fungicides. For example, all *U. necator* isolates with substitution Y136F exhibited high resistance (RF > 5), while isolates lacking mutations were sensitive or weakly resistant (RF < 5)^22^. In the present study, the mutant UV10th generated by UV irradiation contained the Y137H mutation in VvCYP51. The difference in EC_50_ values of transformants pB-Vv51wt and pB-Vv51mut indicated that the Y137H was a DMI resistance determinant. This result was further solidified by molecular docking analysis and VvCYP51 protein-tebuconazole binding assays. Although alterations of amino acid tyrosine at position 137 is a known mechanism of DMI fungicide resistance, the change of tyrosine to histidine at this position has not been reported to the best of our knowledge.

For many pathogens, including *Bulbophyllum jaapii*^14^, *P. digitatum*^11^,^13^,^17^, *M. fructicola*^12^, *Venturia inaequalis*^34^, *Cercospora beticola*^15^, and *M. graminicola*^16^, up-regulation of *CYP51* genes reduced sensitivity to DMI fungicides. Although the *VvCYP51* gene in mutant UV10th was overexpressed, there was no correlation between expression of *VvCYP51* genes and EC_50_ values in 26 pB-Vv51wt transformants. These data indicate that increased expression of the *VvCYP51* gene did not play an important role in conferring reduced sensitivity to DMI fungicide tebuconazole. Similar results were also observed in *Puccinia triticina*^35^. It is still not clear why the *VvCYP51* gene in the mutant UV10th was up regulated; the 1109 bp upstream region of *VvCYP51* gene did not vary compared to the wildtype and no additional promoters or other changes were found that could have increased expression. Nevertheless, we noticed that the expression of the *VvCYP51* gene in all pB-Vv51wt transformants had increased but there was no association (P > 0.05) with the reduced sensitivity.

Molecular modeling of the *CYP51* genes in *M. fijiensis* and *M. graminicola* demonstrated that tyrosine at aa position 136 and 137 was involved with the affinity of CYP51 proteins to DMI fungicides^21^,^36^,^37^. Molecular docking analysis is widely used to assess the binding between proteins and chemicals^15^,^21^,^33^,^36^,^38^. For example, some compounds were demonstrated to interact more tightly with the Hsp90 protein due to higher total scores of binding^39^. Binding energy and inhibition constant calculated by Autodock were also widely used to estimate the extent of binding^40^. In our study, SYBYL software was used to simulate the binding mode of the protein (VvCYP51) and ligand (tebuconazole). There was a stronger binding interaction between tebuconazole and VvCYP51 compared to tebuconazole and VvCYP51/Y137H despite the lack of a hydrogen bond between tebuconazole and Tyr137, suggesting that the point mutation decreased the binding property between the VvCYP51 and tebuconazole. Besides, the binding energy calculated by AutoDock directly showed that the interaction between tebuconazole and VvCYP51 protein was decreased if the protein contained the Y137H mutation.

UV-visible absorption spectroscopy has been widely used in bacteria, fungi, plants, and human for the determination of the binding between fungicides and P450s, and was confirmed to be a simple and accurate method for detection of direct interactions between proteins and fungicides^41^,^42^,^43^,^44^,^45^,^46^. The calculated K_d_ is generally used to assess the affinity between proteins and fungicides. For example, McLean *et al.* found that the binding of clotrimazole (K_d_ < 0.07), econazole (K_d_ < 0.09) and miconazole (K_d_ < 0.10) to *Mycobacterium tuberculosis* CYP121 was much tighter compared to imidazole (K_d_ = 64) and phenylimidazole (K_d_ = 53)^43^. In our study, the *K*_d_ values of three different truncated VvCYP51 proteins (less than 0.04 μM) were significantly lower than that of three truncated VvCYP51/Y137H proteins (more than 0.07 μM) (Table 4), indicating VvCYP51 had higher affinity for tebuconazole than VvCYP51/Y137H. Most CYP51 proteins are water soluble, but not CYP51 from *Magnaporthe grisea*, in which inclusion body is similar to VvCYP51 protein. We tried to express the full length cDNA of the FJ4-1b and UV10th in *E. coli* and *Pichia* strains, however, no predicted molecular mass of VvCYP51 was observed. Therefore, we erased the membrane-spanning area of the VvCYP51 by constructing three different truncations (28-, 42-, 74 aa) of N terminus.

The interaction of different substrates and P450s generally show three spectral binding patterns. The azole fungicides that inhibit P450s show type II binding spectra, which exhibit an absorbance maximum at 430 nm and an absorbance minimum at 410 nm^47^. In our study, the absorbance maximum and minimum were at 395–405 and 375–385 nm, respectively. The blue shift in the spectra may have been caused by other membrane-binding components. This phenomenon was also reported by Xiao *et al*^45^.

In conclusion, our study shows that the Y137H mutation in the *VvCYP51* gene was responsible for the reduced sensitivity to the DMI fungicide tebuconazole. This result was supported by genetic transformation experiments, analysis of interaction between VvCYP51 and tebuconazole through both bioinformatic analysis and experimental binding assay.

## Materials and Methods

### *V. virens* isolates used in this study

The single spore isolate UV-8a used in previous study^48^, was used to clone the *V. virens CYP51* (*VvCYP51*) gene. Isolate FJ4-1b was isolated from Fujian province of China in 2009 and used for ultraviolet (UV) mutagenesis and transformation based on its ability to grow and sporulate profusely on potato sucrose agar (PSA).

### Cloning and sequence analysis of *VvCYP51* gene

Genomic DNA of the isolate UV-8a was extracted from mycelium using the EasyPure Plant Genomic DNA Kit (TransGen Biotech, Beijing, China) according to the manufacturer’s recommendation. An internal fragment of the *VvCYP51* gene were amplified with a pair of degenerate PCR primers AJ235 and AJ236^49^. Amplification reactions were performed in a 50 μl reaction volume containing 2.5 units of Easy Taq DNA polymerase (TransGen Biotech, Beijing, China), 1×PCR reaction buffer provided by the manufacturer, 200 μM of each dNTP, 0.4 μM of each primer and 50 ng of template DNA. Amplification was carried out in a Mycycler thermal cycler (Bio-Rad Laboratories Inc., California, USA) with the following profile: 94 °C for 3 min, 30 cycles at 94 °C for 30 sec, 45 °C for 1 min, 72 °C for 1 min, and a final extension at 72 °C for 5 min.

The complete *VvCYP51* sequence and flanking sequences were obtained by successive inverse PCR (IPCR) amplifications. Primers are listed in Table S1. Amplification reactions were performed in a 50 μl reaction volume largely similar with that described in amplifying internal fragment of the *VvCYP51* gene except for with the following profile: 94 °C for 3 min, 35 cycles at 94 °C for 40 sec, 59 °C (VvCYP51-F1-R1, VvCYP51-F2-R2, VvCYP51-F5-R5) or 46 °C (VvCYP51-F3 -R3, VvCYP51-F4 -R4) for 40 sec, 72 °C for 3 min, and a final extension at 72 °C for 5 min. All amplified PCR products were purified with a gel extraction kit (TransGen Biotech, Beijing, China), then ligated into the pMD 18-T vector (Takara, Dalian, China), and sequenced with the vector primers at Beijing Genomics Institute (BGI, Shenzhen, China). The DNASTAR software (DNASTAR Inc., Nevada City, CA) was used to assemble and align the *VvCYP51* sequence.

The protein sequences of related fungal *CYP51* genes were obtained from the NCBI GenBank, and phylogenetic analysis was performed with the maximum likelihood (ML) method using MEGA 5.2 software^50^. The following settings were used: heuristic search using close neighbor interchange (CNI; level = 1) with initial trees generated by random addition (100 reps).

### UV irradiation

The wild type isolate FJ4-1b was grown in 50 ml of potato sucrose broth (PSB), started with three mycelial plugs per flask taken from actively growing colonies. The flasks were incubated at 28 °C for 7 days and rotated on a 160 rpm orbital shaker. The broth containing hyphae and conidia was filtered through double sterile gauzes from the PSB culture to separate hyphae from conidia. Then 1 ml of the conidial suspension (10^6^ conidia/ml) was placed in a sterilized 6 cm petri dish and exposed to UV radiation with 90 μw/cm^2^ intensity at 30 cm under UV light for 2 min. Approximately 0.2 ml of the treated suspension was spread on one 9 cm PSA plate. After 5 days of incubation at 28 °C in darkness, about 20 ml of PSA amended with 0.5 μg/ml tebuconazole was poured onto the plate. The growing colonies were transferred to fresh PSA for adaptation incubation. Seven days later, the mycelia were transferred to PSA with 0.5 μg/ml tebuconazole to confirm the reduced sensitivity.

### RNA extraction, cDNA synthesis, and quantification of *VvCYP51* expression

The wild type isolates, mutant, and transformants were grown in 50 ml of PSB at 28 °C for 7 days on a 160 rpm orbital shaker. Total RNA was extracted from mycelia using the TRIzole reagent (Aidlab, Beijing, China), then digested with DNase I nuclease (Takara, Dalian, China) to remove DNA contamination. The cDNA was synthesized using a RevertAid First Strand cDNA Synthesis Kit employing the oligo(dT)_18_ primer (Thermo Fisher Scientific, Waltham, MA). The expression of the *VvCYP51* gene was evaluated by real-time PCR using the primer pair RealCYP51-F/RealCYP51-R. Expression was normalized with the α-tubulin gene. Relative expression was calculated using the comparative C_t_ (2^−ΔΔCt^) method^51^. In order to determine whether the expression of *VvCYP51* gene is inducible by tebuconazole, the expression of *VvCYP51* gene in wild type isolate FJ4-1b was investigated after exposure to 0.1 μg/ml tebuconazole for 6 h. Real-time PCR amplifications of *VvCYP51* were performed in a CFX96 Real-Time PCR Detection System (Bio-Rad Laboratories Inc., Hercules, CA) using SYBR Green I fluorescent dye. Amplifications were conducted in a 20-μl volume containing 10 μl of SYBR qPCR Mix (Aidlab, Beijing, China), 1 μl of RT product and 0.2 μM of each primer. All samples were in triplicates with the following profile: an initial preheat at 95 °C for 2 min, followed by 40 cycles at 95 °C for 20 s, 60 °C for 20 s and 72 °C for 20 s. The entire experiment starting with RNA extractions was repeated four times.

### Construction of expression vectors of the wild type and mutated *VvCYP51* alleles

A 2956 bp fragment containing the full-length *VvCYP51* with point mutation, a 1109 bp upstream flanking sequence, and 20 bp downstream flanking sequence of the mutant UV10th was amplified with primers KpnI-F/KpnI-R (Fig. 1), which contained *Kpn*I restriction sites. Amplification reaction was performed in a 50 μl reaction volume containing 1.25 unit of PrimeSTAR HS DNA polymerase, 1×PrimeSTAR buffer provided by the manufacturer, 200 μM of each dNTP, 0.4 μM of each primer and 50 ng of template DNA. Amplification was carried out in a Mycycler thermal cycler (Bio-Rad Laboratories Inc., California, USA) with the following profile: 94 °C for 3 min, 35 cycles at 94 °C for 40 sec, 59 °C for 40 sec, 72 °C for 3 min, and a final extension at 72 °C for 5 min. PCR product was purified with a gel extraction kit (TransGen Biotech, Beijing, China). The purified product was digested with *Kpn*I and cloned into the vector pBHt2, creating a vector designated pB-Vv51mut. The corresponding fragment was amplified from wild type isolate FJ4-1b and cloned into pBHt2 as control (pB-Vv51 wt). Plasmid inserts were sequenced for quality control before being transformed into the *Agrobacterium tumefaciens* strain EHA105 by electroporation.

### Transformation of *V. virens*

The *E. coli* strain Trans5α (TransGen Biotech, Beijing, China) was used for plasmid construction and propagation in Luria-Bertani (LB) broth or on LB plates supplemented with ampicillin (100 μg/ml), kanamycin (50 μg/ml) when required. The *A. tumefaciens* strain EHA105 was used for transformation and grown in LB broth or on LB plates supplemented with kanamycin (50 μg/ml), rifampicin (50 μg/ml), streptomycin (50 μg/ml) when required. All strains were stored at −70 °C in 25% glycerol.

The method used for transformation of *V. virens* was performed as described before with minor modifications^52^. Conidia suspensions were prepared as described in the UV irradiation section. To co-cultivate *V. virens* and *A. tumefaciens*, the spore concentration was adjusted to 10^6^ spores/ml. An aliquot of 200 μl of spore-bacterial cell mixture was spread onto a cellophane membrane on the co-medium containing 400 μM of acetosyringone. After incubation at 28 °C for 6 days, the membrane was transferred to a sterile empty plate, then covered with PSA containing 200 μg/ml of cephalosporin to suppress bacteria, and 200 μg/ml of hygromycin to select for *V. virens* transformants. After incubation at 28 °C for 8 to 10 days, the transformants were transferred to fresh PSA plates containing 200 μg/ml of hygromycin for a second round of selection.

### Determination of tebuconazole sensitivity

Sensitivity of *V. virens* UV-8a, UV10th and transformants to tebuconazole (96% active ingredient, Bayer Crop Science, Leverkusen, Germany) was determined by calculating the 50% inhibitory dose (EC_50_ value) on PSA dishes amended with 0, 0.01, 0.03, 0.1, 0.3, and 1 μg/ml tebuconazole. Mycelial plugs (6 mm) were transferred in triplicates from the margins of 14-day old colonies to 9 cm petri dishes containing nonamended or tebuconazole-amended PSA. Dishes were incubated at 28 °C for 14 days in darkness. The average colony diameter was measured in two perpendicular directions, and the EC_50_ value was determined by regressing percentage growth inhibition against the log of fungicide concentration.

### Determination of mycelial growth, sporulation and spore germination

The wild type isolate, mutant, and transformants used in this study were tested for mycelial growth rate, sporulation and spore germination. Three 6 mm mycelia PSA plugs for each isolate were transferred to the center of three PSA plates for radial growth measurements. After incubation at 28 °C for 14d in the dark, the colony diameter of each isolate was measured. To determine conidial production, all the isolates were grown in 50 ml of PSB at 28 °C on a 160 rpm orbital shaker. After 10 days, the conidial suspension was filtered to the 6 cm petri dish by double sterile gauze from PSB culture, then the suspension was mixed by tips and the sporulation was assessed with a haemocytometer under a microscope. To determine the spore germination, the 200 μl conidial suspension from 7d PSB culture was spread onto PSA medium. After 12 h incubation at 28 °C in the dark, 100 conidia per isolate were assessed under a microscope. Each isolate was tested in triplicates.

### Molecular docking analysis

Structural modeling of *V. virens* CYP51 (VvCYP51) from wild type isolate FJ4-1b and the mutant UV10th was carried out using the Swiss-Model interface with a resolution of 1.90 Å^53^. Molecular docking analysis was performed using molecular modeling software package SYBYL-X 2.0 (Tripos Inc., St. Louis, USA). The protomol (idealized active site) was generated from hydrogen-containing protein mol2 file by keeping the default parameters (threshold factor of 0.5 Å and a bloat of 0 Å)^54^. The molecular structure of ligand (tebuconazole) was obtained through NIST Chemistry WebBook (http://webbook.nist. gov/chemistry), and converted into mol2 file format using Discovery Studio Client 2.5 (Accelrys Inc., San Diego, CA). Then the ligand was prepared using ‘docking >1_conformation’ protocol and docked with the prepared protein at the developed protomol.

The binding energy between tebuconazole and VvCYP51 protein was calculated using AutoDock tools (ADT) v 1.5.6^55^ and Autodock v 4.2 programs (Autodock, Autogrid, the Scripps Research Institute, San Diego, CA). Based on molecular docking analysis, active site LYS148 was selected as flexible amino acid for wild type VvCYP51, while HIS137 and ARG379 were selected for VvCYP51/Y137H. Grid box was set as 100×100×100 Å^3^, and grid spacing was kept as default 0.375 Å. The ligand was prepared adding Gasteiger charges, polar hydrogen and keeping rotatable bond maximum of 6. The best docking result was considered as the conformation with the lowest binding energy (∆G)^56^.

### Heterologous expression of VvCYP51 in *E. coli*

N-terminal 74 amino acid (aa) region in VvCYP51 was predicted to be membrane-spanning leader sequence by TMHMM (http://www.cbs.dtu.dk/services/TMHMM). In order to heterologously express the *VvCYP51* gene, the membrane-spanning leader sequence should be removed. Thus three different truncations (28-, 42- and 74 aa) of N terminus of VvCYP51 which showed no membrane-spanning area by TMHMM were developed. The *VvCYP51* genes that generate 28-, 42- and 74 aa truncation of N-terminal amino acid were amplified with primers Hind-R and Bgl-F-28, Bgl-F-42, Bgl-F-74 from cDNAs of the FJ4-1b and UV10th, respectively. The truncated *VvCYP51* genes were digested with *Hin*dIII and *Bg*lII, and ligated into the T7 lac promoter vector pET28a (or pET32a) that had been pre-digested with *Hin*dIII and *Bam*HI. All plasmids were sequenced to ensure the correctness of the sequences and transformed into *E. coli* DE3 competent cell (TransGen Biotech, Beijing, China). The transformants were grown in 1.0 L LB broth containing 100 μg/ml ampicillin (pET32a) or 50 μg/ml kanamycin (pET28a) at 37 °C with 200 rpm shaking until the OD values were 0.2 to 0.6. Then isopropyl β-D-thiogalactopyranoside (IPTG) was added to a final concentration of 0.5 mM and incubated at 18 °C for 4 h. Cells were harvested by centrifugation at 5000 g for 5 min, washed by PBS buffer (8 g NaCl, 0.2 g KCl, 3.58 g Na_2_HPO_4_·12H_2_O, 0.27 g KH_2_PO_4_ for 1 L, PH 7.4), and resuspended in 30 ml potassium phosphate buffer (pH 7.4) containing 100 mM K_2_HPO_4_, 100 mM KH_2_PO_4_, 1 mM EDTA, and 20% glycerol. Lysozyme (1 mg/ml), pheylmethylsulfonyl fluoride (PMSF, 1 mM) and dithiothreitol (DTT, 1 mM) were added in the suspension, and disrupted by using a JY92-2D Sonicator (Ningbo Scientz Biotechnology co., Ningbo, China). The lysate was centrifuged at 10000 g for 15 min at 4 °C, and the precipitate was resuspended by potassium phosphate buffer. The total protein concentration was determined spectrophotometrically with Bradford protein assay kit (Aidlab, Beijing, China) according to the manufacturer’s instructions.

### Western blot analysis

In order to evaluate whether the VvCYP51 proteins were successfully expressed, the heterologously expressed products were mixed with loading buffer (TransGen Biotech, Beijing, China) and boiled for 10 min, then separated on a 10% sodium dodecyl sulfate-polyacrylamide gel (SDS-PAGE) and transferred onto a polyvinylidene fluoride membrane (Bio-Rad Laboratories Inc., California, USA). For detection of fusion protein of VvCYP51:His, membranes were probed with a 1:3000 dilution of anti-His tag mouse monoclonal antibody and subsequently a 1:10,000 dilution of goat anti mouse IgG HRP (Beijing ComWin Biotech, Beijing, China). Antibody binding was detected using the clarity ECL western blotting substrate (Bio-Rad Laboratories Inc., California, USA) and the signal from chemoluminescence was captured using ChemiDoc XRS + Imager (Bio-Rad Laboratories Inc., California, USA).

### Binding assay

UV-visible absorption spectra from 350 to 500 nm were recorded for mixtures of 2 ml of VvCYP51 protein at 1 mg/ml and tebuconazole at 0.05, 0.1, 0.15, 0.2, 0.25, 0.75 and 1.25 μM on S3100 UV-visible scanning spectrophotometer (Scinco Inc., Daedeok, Korea) after 1 min of incubation using 1 cm pathlength quartz cells for each tebuconazole concentration. The *K*_*d*_ value for the tebuconazole binding was calculated by Hanes-Woolf plot following the equation: A = A_max_ [I] / (K_d_ + [I]), where [I] is the tebuconazole concentration, A and A_max_ are the observed absorption difference at each concentration of tebuconazole and maximal absorption difference at tebuconazole saturation, respectively.

### Statistical analysis

Significant differences of EC_50_ values, mycelial growth rate, sporulation, spore germination and *K*_d_ values of tebuconazole with wild type and mutant VvCYP51 proteins were evaluated by one-way ANOVA analysis with least-significant-difference test in SPSS for Windows Version 13.0 (SPSS Inc, Chicago, Illinois, USA). Linear association between EC_50_ values of *VvCYP51* transformants (pB-Vv51wt transformants) and relative expression values of the *VvCYP51* gene was examined using the Pearson’s correlation coefficient.

## Additional Information

**How to cite this article**: Wang, F. *et al.* The Y137H mutation of *VvCYP51* gene confers the reduced sensitivity to tebuconazole in *Villosiclava virens*. *Sci. Rep.* **5**, 17575; doi: 10.1038/srep17575 (2015).

## Supplementary Material

Supplementary Table S1

## Figures and Tables

**Table 1:** Relative expression of the *VvCYP51* gene and tebuconazole sensitivity in 26 pB-Vv51wt transformants.

Isolate/transformant	Relative expression of the *VvCYP51* gene^a^	EC_50_ value	Resistance factor
FJ4-1b	1.00	0.04	
UV10th	55.04 ± 13.90	0.22	5.12
P-F 4	3.43 ± 0.82	0.11	2.67
P-F 7	8.70 ± 1.93	0.24	5.79
P-F 8	10.86 ± 6.50	0.13	3.10
P-F 9	20.90 ± 0.32	0.15	3.67
P-F 10	3.49 ± 1.87	0.12	2.90
P-F 13	2.84 ± 1.11	0.11	2.69
P-F 14	4.59 ± 0.58	0.28	6.64
P-F 15	15.74 ± 2.21	0.13	3.02
P-F 16	2.68 ± 1.06	0.13	3.17
P-F 17	4.35 ± 0.52	0.20	4.71
P-F 20	5.55 ± 0.46	0.27	6.50
P-F 22	10.49 ± 1.54	0.15	3.52
P-F 23	24.23 ± 6.76	0.20	4.76
P-F 25	8.25 ± 1.03	0.15	3.64
P-F 26	4.11 ± 2.67	0.18	4.26
P-F 28	3.86 ± 1.60	0.13	3.05
P-F 29	8.45 ± 2.55	0.26	6.07
P-F 31	3.13 ± 1.20	0.25	5.83
P-F 32	6.70 ± 0.03	0.32	7.71
P-F 33	4.14 ± 1.39	0.12	2.74
P-F 34	2.57 ± 1.79	0.15	3.55
P-F 36	9.50 ± 0.83	0.22	5.33
P-F 37	6.43 ± 2.84	0.11	2.52
P-F 38	5.76 ± 3.91	0.31	7.29
P-F 39	5.53 ± 2.33	0.13	2.98
P-F 40	6.88 ± 0.13	0.21	4.90

^a^The expression of the *VvCYP51* gene was normalized using α-tubulin gene expression levels and then compared to *VvCYP51* expression in FJ4-1b. Data are shown as mean values ± standard errors.

**Table 2:** Mycelial growth rate, sporulation and spore germination of all *V. virens* isolates used in this study.

Isolate/transformant	Description	No. of isolates	Colony growth rate (mm d^−1^)		Sporulation^a^		Spore germination^b^	
			Mean^c^	Range	Mean^c^	Range	Mean^c^	Range
FJ4-1b	Wild type isolate	1	3.9 ± 0.34ab	3.5–4.3	5.7 ± 0.22a	5.4–5.8	90.0 ± 2.00a	88.0–92.0
UV10th	UV mutagenesis mutant	1	3.4 ± 0.11b	3.3–3.5	6.5 ± 0.04a	6.4–6.5	81.3 ± 6.11a	76.0–88.0
P-F	pB-Vv51wt transformants	26	2.7 ± 0.56c	1.4–3.3	4.8 ± 2.33a	0–7.1	78.0 ± 22.29a	32.0–97.3
P-10th	pB-Vv51mut transformants	13	4.0 ± 0.60a	3.1–4.7	2.1 ± 2.08b	0–4.8	76.0 ± 22.52a	50.0–89.3

^a^log-transformed number of conidia per cm^2^.

^b^Percentage of germinated conidia after 12 h incubation (n = 100).

^c^Mean ± S.E.M (standard error of mean); values within the same column followed by the same letters are not significantly different based on the analysis of least significant difference (LSD) test at *P* = 0.05.

**Table 3:** Docking results of the CYP51-tebuconazole complex.

	Total score	Crash	Polar	Distance of centroids	Binding energy (kcal/mol)	Inhibition constant (uM)
FJ4-tebuconazole	5.4982	−1.5651	0.1136	6.649	−6.93	8.27
UV10th-tebuconazole	4.4795	−0.7135	2.1472	6.898	−6.63	13.72

Total score: total output of all the scores. Crash: the ability of the compound to penetrate the active site of the protein. Polar: the polar interaction between the ligand and the protein. Inhibition constant: the ligand concentration when the ligand inhibited 50% protein activity.

**Table 4:** *K*_d_ values (μM) of tebuconazole with three different truncated wild type VvCYP51 proteins and 3 truncated VvCYP51/Y137H.

	−28aa	−42aa	−74aa
VvCYP51	0.037 ± 0.0027	0.034 ± 0.0023	0.037 ± 0.0025
VvCYP51 with Y137H	0.081 ± 0.0070	0.070 ± 0.0021	0.075 ± 0.0024
