# Supplementary material for: The Y137H mutation of VvCYP51 gene confers the reduced sensitivity to tebuconazole in Villosiclava virens
Source: Sci Rep. 2015 Dec 3;5:17575. doi: 10.1038/srep17575 (PMC4668384; doi:10.1038/srep17575)
Supplement: Supplementary Table S1 [file srep17575-s1.doc]

**Supplementary File**

**The Y137H mutation of *VvCYP51* gene confers the reduced sensitivity to tebuconazole in *Villosiclava virens***

**Fei Wang^1^, Yang Lin^1^,** **Wei-Xiao Yin****^1^, You-Liang Peng^2^,** **Guido Schnabel^3^**, **Jun-Bin Huang^1^ and Chao-Xi Luo^1*^**

**^1^**Department of Plant Protection, College of Plant Science and Technology and the Key Lab of Crop Disease Monitoring & Safety Control in Hubei Province, Huazhong Agricultural University, Wuhan 430070, China;

**^2^**Department of Plant Pathology, College of Agriculture and Biotechnology, China Agricultural University, Beijing 100193, China;

**^3^**Department of Agricultural and Environmental Sciences, Clemson University, Clemson, SC 29634, USA

***Table S1*** Nucleotide sequence and characteristics of primers used in this study

| Primer | Sequence(5’→3’) | Description | Source or reference |
| --- | --- | --- | --- |
| AJ235 | GARCCACCWGTTGTMTTTCA | Degenerate primer for *VvCYP51* amplification | 33 |
| AJ236 | GTASTYYTCTTCGGCGTTGAC | Same as for AJ235 | 33 |
| VvCYP51-F1 | GGAACAGGGTAAAGGACGTT | Inverse PCR primer for *VvCYP51* amplification | This study |
| VvCYP51-R1 | CTGTTCGCTTACGTTCGTTT | Same as for VvCYP51-F1 | This study |
| VvCYP51-F2 | TGCCATATGTAATGGTGCTG | Same as for VvCYP51-F1 | This study |
| VvCYP51-R2 | TCTCTGCCGAAGTACAATCC | Same as for VvCYP51-F1 | This study |
| VvCYP51-F3 | GCATTGGGAGAGTCTGCTTC | Same as for VvCYP51-F1 | This study |
| VvCYP51-R3 | TTGTCCGTCTGTTGAAGCTG | Same as for VvCYP51-F1 | This study |
| VvCYP51-F4 | AAAGGGATGACATAGGGTCT | Same as for VvCYP51-F1 | This study |
| VvCYP51-R4 | ATCTGAATGGTCGTCAACCT | Same as for VvCYP51-F1 | This study |
| VvCYP51-F5 | CCAAGTTCTACTGTCTGCAA | Same as for VvCYP51-F1 | This study |
| VvCYP51-R5 | ATCTGAATGGTCGTCAACCT | Same as for VvCYP51-F1 | This study |
| RT-F | ATGGGCGTCCTTCAAGACGT | RT-PCR primer for amplifying the complete coding sequence of *VvCYP51* | This study |
| RT-R | CTAATCTCGTCGTTCCCAGA | Same as for RT-F | This study |
| RealCYP51-F | GAGGAGGAAAAGGTCGACTA | Real-time PCR primer for amplifying a 182-bp fragment of *VvCYP51* | This study |
| RealCYP51-R | CTATTGCCACCATCAACATT | Same as for RealCYP51-F | This study |
| α-tubulin-F | GGCGTTTACAATGGCACTTC | Real-time PCR primer for amplifying a 158-bp fragment of *Vvtubulin*, the endogenous control | This study |
| α-tubulin-R | CGGAACAGTTGACCAAAAGG | Same as for α-tubulin-F | This study |
| KpnI-F | GGGGTACCAGTAACGAAACAGCCACT | Construction of pBHt2-Vv51wt and pBHt2-Vv51mut expression plasmid; restriction sites for KpnI (underlined) were added | This study |
| KpnI-R | GGGGTACCTCGACTCTCCAGTCCCTAAT | Same as for KpnI-F | This study |
| Hind-R | CCCAAGCTTCTAATCTCGTCGTTCCCAGAAAA | Construction of heterologous expression plasmid; restriction sites for HindIII (underlined) were added | This study |
| Bgl-F-28aa | GAAGATCTTTGACCGCCTTCGTTGTT | Same as for Hind-R; restriction sites for BglII (underlined) were added | This study |
| Bgl-F-42aa | GAAGATCTAACCAGTTTCTCTTTGCG | Same as for Hind-R | This study |
| Bgl-F-74aa | GAAGATCTAAGTTCTTGCATGAAAAT | Same as for Hind-R | This study |
|  |  |  |  |
